# Family Caregivers' Experiences of Involuntary Psychiatric Hospital Admissions of Their Relatives – a Qualitative Study

**DOI:** 10.1371/journal.pone.0025425

**Published:** 2011-10-12

**Authors:** Jelena Jankovic, Ksenija Yeeles, Christina Katsakou, Tim Amos, Richard Morriss, Diana Rose, Peter Nichol, Rosemarie McCabe, Stefan Priebe

**Affiliations:** 1 Unit for Social and Community Psychiatry, Barts and the London School of Medicine and Dentistry, Queen Mary University of London, London, United Kingdom; 2 Academic Unit of Psychiatry, University of Bristol, Bristol, United Kingdom; 3 School of Community Health Sciences, University of Nottingham, Nottingham, United Kingdom; 4 Institute of Psychiatry, King's College London, London, United Kingdom; The University of Melbourne, Australia

## Abstract

**Background:**

Family caregivers of people with mental disorders are frequently involved in involuntary hospital admissions of their relatives.

**Objective:**

To explore family caregivers' experience of involuntary admission of their relative.

**Method:**

30 in-depth interviews were conducted with family caregivers of 29 patients who had been involuntarily admitted to 12 hospitals across England. Interviews were analysed using thematic analysis.

**Results:**

Four major themes of experiences were identified: relief and conflicting emotions in response to the relative's admission; frustration with a delay in getting help; being given the burden of care by services; and difficulties with confidentiality.

Relief was a predominant emotion as a response to the relative's admission and it was accompanied by feelings of guilt and worry. Family caregivers frequently experienced difficulties in obtaining help from services prior to involuntary admission and some thought that services responded to crises rather than prevented them. Family caregivers experienced increased burden when services shifted the responsibility of caring for their mentally unwell relatives to them. Confidentiality was a delicate issue with family caregivers wanting more information and a say in decisions when they were responsible for aftercare, and being concerned about confidentiality of information they provided to services.

**Conclusion:**

Compulsory admission of a close relative can be a complex and stressful experience for family caregivers. In order for caregivers to be effective partners in care, a balance needs to be struck between valuing their involvement in providing care for a patient and not overburdening them.

## Introduction

The importance of family caregivers' (in the literature also referred to as ‘carers’) involvement in mental health services has been increasingly emphasised over the past decade and there is a wide consensus that family caregivers should be seen by clinicians as partners in the care of patients [1]. Various policy documents support this move, for example in England a National Service Framework [2] addresses the needs of caregivers.

However, evidence suggests that family caregivers' experiences of working with mental health professionals are mixed. Some family caregivers experience distress as a result of their interaction with the mental health care system (i.e. in negotiating crisis situations; acting as advocates for patients, dealing with legal barriers etc. [3]) whereas others are satisfied with their involvement [4]. A potential discrepancy between the wishes of family caregivers and patients can present an additional complication. Research suggests that family caregivers often request more supportive and intensive interventions, whereas patients prefer treatment options preserving their autonomy and independence [5].

The burden that family caregivers experience refers to the physical, psychological and social impact that caring for relatives with chronic disorders has on families [6], [7]. A distinction has been suggested between objective burden (including costs to the family such as disruption of everyday life) and subjective burden (including individual's perception of the situation as burdensome [8], [9]). Their role may impact on caregivers' physical and mental health as well as on their emotional wellbeing [10]. In practice, the needs of family caregivers tend to be poorly recognised both by clinicians and by patients [11], and they feel marginalised by services [12].

Involuntary admission to hospital and subsequent treatment against the patient's will can be stressful for all parties involved, including family members who are caring for the patient. Whilst several studies assessed patients' experience of involuntary psychiatric admissions, the experiences and views of family caregivers remain poorly understood [13], . A better understanding of the experiences of family caregivers may help address their needs more effectively and improve their involvement in care during and after an involuntary hospital admission.

### Aim

This study aimed to explore how family caregivers of patients who were involuntarily admitted to a psychiatric hospital experience the involuntary admission and the subsequent hospital treatment of their relative.

## Materials and Methods

In this qualitative research study, in-depth semi-structured interviews were carried out with family caregivers of patients who were involuntarily admitted to 12 hospitals across England run by the National Health Service (NHS) organisations and interviews were part of a larger national multi-centre study on Outcomes of Involuntary Hospital Admission in England. Details of the study and findings on patient outcomes have been described elsewhere [15], [16]. The NHS is publicly funded healthcare system in the UK that is free at the point of use. Mental health services for adults in the NHS are structured into community teams (including crisis teams and specialised teams) and inpatient services. The study was carried out before Community Treatment Orders [17] were introduced into mental health legislation in England.

A family caregiver was defined as 1) a partner or relative who lives with the patient or visits/meets him/her at least three times a week and 2) who has a role in the care of the patient.

### Sampling and data collection

Purposive sampling was used to maximise the likelihood of obtaining a complete range of views and included: family caregivers with different relationships to the patients (i.e. parents, partners, siblings, children); family caregivers of patients with and without previous hospital admissions; and family caregivers of patients of different ethnic origin.

Family caregivers were initially contacted by letter or telephone after patients recruited in the national study provided informed written consent to approach their family caregivers. Written consents to contact family caregivers were obtained during the 3 or 12- months follow-up structured interviews. As ethical approval was granted after a national study was already under way, patients who already completed their participation were not contacted. Recruitment proved to be difficult as majority (77%) of patients interviewed at 12 months follow up were living alone and majority of them did not have a family caregiver [15]. At one study site the number of reported family caregivers was very small and participating patients were approached again in order to identify any additional family caregivers. The majority of interviews were conducted at the interviewee's home.

The study has received ethical approval from the UK Multi-Centre Research Ethics Committee (ref. MREC/03/0/96). In all centres where participants were interviewed (University of Bristol, University of Liverpool, King's College University of London and Queen Mary University of London and their associated Mental Health NHS Organisations) written informed consent was obtained from all participants.

### Topic guide - interviews

The interviews focused on the specific experiences of family caregivers related to the index involuntary admission of their relative (the admission that led to including patients into the original study) rather than views about services or involuntary treatments more generally. Participants were asked to describe events related to this index admission in order to help them focus on this admission specifically.

A topic guide for the interviews was designed and finalised between researchers and a family caregivers' representative. It covered family caregivers' experiences and evaluation of their involvement immediately before, during and after involuntary admission of their relative as well as their emotional responses. Further details on main interview topics are presented in Table 1.

**Table 1:** Main topics for qualitative interview.

*Before admission*
What led to your relative being admitted against their will?
How was your relative feeling/behaving?
What did you do about it?
How did this affect your life?
*Procedure of admission to hospital*
Who was involved in the process of involuntary admission?
Were you involved in this?
How did you feel about it?
How did it affect your life/relationship?
How do you feel about it now?
*Hospital admission*
What was the hospital like (including healthcare professionals?)
Were you involved in a care planning?
How did you feel about relative's treatment?
How did you feel when your relative was discharged?
How was your relationship/life together after their discharge?
Do you think anything has changed as a result of your relative being compulsory treated?

### Data analysis

A family caregiver from a local carers' organisation was involved in designing the list of topics for the interview. Another caregiver (not interviewed in the study) whose close relative had been involuntarily admitted was involved in the analysis of the data and preparation of the manuscript and is a co-author (PN).

All interviews were audio-recorded and transcribed verbatim. Thematic analysis was used for identification of themes. MAXqda software (Version 2) for qualitative data analysis was used to aid the coding. The transcripts were then analysed thematically, following the stages of open and selective coding described in grounded theory. A coding frame capturing the emerging themes was devised by two researchers (JJ and KY) and further discussed and refined in team meetings which included a family caregiver. The coding frame was then elaborated and modified as new themes and subthemes emerged in the course of the analysis [18]. As part of the analysis we also looked at deviant cases. The final definition of themes was reached in a series of team discussions. After initial coding of the data, all of the data exemplifying each theme was re-examined to further check its' consistency with that theme. To examine inter-rater reliability of the coding, two researchers independently coded all interviews (JJ and KY), compared their results and a consensus on final coding was reached through discussion involving both coders and other members of the team (SP, CK, RMc). The members of the research team had different backgrounds. PN is a family caregiver; JJ, TA, RM and SP are clinical academic psychiatrists; KY and RMC are research psychologists; CK is a research psychologist with clinical experience; and DR is a social scientist and a mental health service user.

## Results

Thirty interviews with 31 family caregivers were analysed (12 male and 19 female family caregivers). Twenty seven patients had one family caregiver interviewed whilst parents of one patient requested to be interviewed together and both parents of another patient were interviewed separately. Therefore 31 family caregivers of 29 patients were interviewed. Out of 31 family caregivers, 16 were parents, seven partners, four siblings, two children, one grandmother and one elderly relative. Twelve patients, whose family caregivers were interviewed, had no psychiatric hospital admissions prior to the index involuntary admission. Twenty one patients were of White ethnic origin, whilst others were of Asian, Black and mixed origin. Four patients were initially admitted to psychiatric hospital as voluntary patients but this was later changed to an involuntary admission. Characteristics of family caregivers and patients are described in Table 2.

**Table 2:** Participant's characteristics.

Patient's age	N	%
18–39	19	66
40–59	9	31
Missing	1	3
**Patient's gender**		
Female	8	28
Male	21	72
**Family caregiver's gender**		
Female	19	61
Male	12	39
**Family caregiver's relationship with patient**		
Parent	16	52
Partner	7	23
Sibling	4	13
Children	2	6
Grandmother	1	3
Elderly relative	1	3
**Number of family caregivers living with patient at the time of admission**		
Yes	23	74
No	8	26
**Patient's ethnicity**		
White	21	72
Asian	4	14
Black	1	3
Mixed	2	7
Missing	1	3
**Whether patient had previous psychiatric hospitalisation**		
Yes	15	52
No	12	41
Missing	2	7

Diagnosis on discharge was Schizophrenia for eight patients, Bipolar Affective Disorder for six, Other Psychotic Disorder for seven, Recurrent Depressive Disorder for two, Schizoaffective Disorder for one, Manic Episode for one, Borderline Personality Disorder for one, and No mental illness for one patient. Diagnosis on discharge was not available for two patients.

### Four themes were identified and are presented below

#### 1. Relief and conflicting emotions in response to the admission (N = 17)

The most common emotional response to the admission was relief (17 family caregivers), in some cases accompanied by other emotions such as worry (5 family caregivers) and guilt (4).

The relief appeared associated with the burden that family caregivers had experienced before the admission.

*The fact that he could stay there for several weeks, it gave us such a lot of relief that he wasn't here.*

*(ID 006, caregiver to a husband, not 1^st^ admission)*

Some family caregivers felt worried and guilty at the same time as relieved, indicating a complex and somewhat conflicting emotional response.

*I felt sick, I really felt sick. I didn't want that to happen, as much as I knew that it was going to make him better, I didn't want him to be, I didn't want him in hospital at all… I was relieved knowing that something was finally being done, because like I said, it was a long process, it was a very long process.*

*(ID 016, caregiver to a brother, 1^st^ admission)*

*So in the end it was kind of relief that somehow you know neither of us had been harmed or (name of the patient) hadn't been harmed but it was pretty close... and I've gone back over it many times, and you know I think well I could have, I should have seen it... I felt guilty that I had let it get to that stage but it was only 3 days, so I couldn't keep up with it.*

*(ID 003, caregiver to a wife, 1^st^ admission)*

We further explored whether family caregivers experienced other emotions unrelated to relief (as part of a deviant case analysis) and it showed that one family caregiver experienced anger in response to their relative's admission.

#### 2. Frustration with a delay in getting help (N = 18)

Many family caregivers stated that mental health services had acted with a delay prior to the involuntary admission, and that they did not know whom to contact to get help. Family caregivers often thought that the delay in response of services contributed to the deterioration of their relative's condition and made the subsequent involuntary admission inevitable. Even when services became involved, they were perceived as not keeping to the agreed schedule of appointments.

*I do wonder if it should have been suggested (for the patient) to be admitted earlier; not allowed to get to the stage he was in… the Crisis Team didn't tend to keep their promises, again they were very busy, but if they said they were coming out and they didn't come, that was such a let down*

*(ID 004, caregiver to a husband, 1^st^ admission)*

*Terrible, trying to get help, absolutely terrible. For week upon week I was trying to get help. To get me brother help. Erm, they'd come, I'd say, try on a Saturday early morning as I can have him in my house, by the time they'd decided to come (name of the patient) was gone. This was going on for weeks and by then (name of the patient) was getting higher and higher and higher. I'd have no sleep whatsoever – for at least I'd say five weeks. We got no help, none whatsoever.*

*(ID 023, caregiver to a brother, not 1^st^ admission)*

Some thought that services focused on responding to adverse events rather than on preventing them and therefore perceived services as reactive rather than proactive.

*The mental health system was good at reaction, but hopeless at pro-action, and unless there was some serious incident, like the self-harming, or the time when she wouldn't get out of bed, to which they could respond with sort of blue lights flashing and so on, they were absolutely useless.*

*(ID 009, caregiver to a wife, 1^st^ admission)*

Family caregivers also reported that they did not know whom to contact to ask for help when their relative was becoming unwell and described these situations as very stressful as they had to look after a relative who was unwell and try to find out how to get help at the same time. Often they were directed from one service to another, without being given clear guidance. This was particularly the case for family caregivers of patients who had no previous hospital admission and were not familiar with the organisation of the mental health system.

*I mean one day he had me in tears, I had to walk out of the house and I just walked into the police station and I spoke to somebody on the desk, and they gave me a little bit of advice and they told me who to contact and stuff, and the next day I rang, I actually spoke to somebody but even that was a long process. I phoned them one day and they said they would get back to me and I said like, I need help now not like tomorrow or next week. I think like they got back to me three months later, it was really, really hard to get any kind of help to start with*

*(ID 016, caregiver to a brother, 1^st^ admission)*

When exploring views of family caregivers who reported a prompt response from services as part of a deviant case analysis, only two experienced a prompt response. In both cases patients had been previously admitted to a psychiatric hospital and had been in contact with services. This low number may not be representative as those who had previous contact with services, may have received appropriate help at an early stage and therefore not needed a hospital admission.

#### 3. Being given the burden of care by services (N = 8)

Some family caregivers reported that services rely on them to take over part of the mental health professionals' role in looking after patients when they are acutely unwell. They believed that too much responsibility was placed on them and stated that they had not been fully consulted about treatment decisions, but were rather implicitly expected to take responsibility for the further care. Not accepting this responsibility usually meant more restrictive options for the patient, e.g. being admitted to hospital or staying longer in the hospital. Family caregivers often believed that their relative was more unwell than judged by the clinicians and that the patient needed more support from clinicians and services than was being offered.

*We were shattered … I didn't really want him to come and spend the night at home already, and one day I went in and it took me completely by surprise Dr X wanted him released that day, and I think that (name of the patient) had only just had his first weekend at home… He (name of the patient) was being really bolshy and still very argumentative, and I said you know perhaps we could just sit quietly and have some time and he was being really horrible…And I really knew I wasn't ready to have him home, but it was really obvious that the doctor wanted him to come home and thought that he was well, and he came home.*

*(ID 006, caregiver to a husband, not 1^st^ admission)*

This disagreement with the clinicians' assessment regarding the level of professional support required was noticeable before discharge and it contributed to the burden of care shifted from services. When that hypothesis was checked in family caregivers who experienced shifting of burden of care prior to hospital admission, it transpired that they also disagreed with the clinician's assessments. More specifically, family caregivers commonly believed that the patient should have been admitted earlier or discharged later, and this was a concern reported mainly by family caregivers of patients with previous hospital admissions.

*I've been begging them to go and section him and they've gone in and assessed him and they've said that he's perfectly alright, and it's been absolute rubbish … I'm put through hell for like 2 weeks, and eventually he is so ill he's sectioned and if they'd only listened to me, like a fortnight beforehand, they could have had him in hospital, done something for him rather than keep calling people out to check him and getting phone calls no sorry in our opinion he's not sectionable, and then 10 days later they section him.*

*(ID 001, caregiver to a son, not 1^st^ admission)*

There was also an example of a family caregiver refusing to take burden of care.

*Um, I've been quite involved…This time we actually refused to have him home because we know that he needs to manage the illness, look after himself…we stuck our necks out and said no you are not coming home, because he was actually causing a lot of stress at home.*

*(ID 027, caregiver to a son, not 1^st^ admission)*

In addition to the burden of looking after a mentally unwell relative who was discharged earlier or admitted later than the family caregivers thought was appropriate, they also reported demands for care during the admission e.g. taking patient on leave when they were not ready and looking for a patient who went missing.

#### 4. Difficulties with confidentiality (N = 7)

Family caregivers repeatedly raised problems relating to confidentiality and lack of information. They understood that confidentiality was a delicate issue, but sometimes considered that if they were providing care for the patient they needed to know relevant information. This information was important in protecting family caregivers from risks but also in allowing them to optimise the level of care.

*Before they will talk to me about anything, they always say is it alright if I talk to your mother which is fine because it's patient confidentiality. But you know, when I'm the one that's at risk, I expect a bit of a say so in it. That's fine if you've got him a safe place and he's being looked after, but when he's out in the community with me, then I expect a say in what goes on*

*(ID 001, caregiver to a son, not 1^st^ admission)*

*And I did keep stressing this to them, 'We're in this as a family’, (name of the patient) is not on his own with this, it involves, it has involved his family for so many years … Because sometimes (name of the patient) would come back from a meeting or he'd go for a group meeting or something and he didn't want to talk and I didn't feel that was doing him any good... while we have very good doctors I would like to be able to go and discuss it and get help from a GP without actually breaking confidence of the patient, I would like to be able to be given a route that we could go through.*

*(ID 025, caregiver to a son, 1^st^ admission)*

Also relating to the issue of confidentiality, family caregivers wanted to provide important information to the clinicians but were concerned that the patient would be told about it, which could in turn adversely affect their relationship.

*I don't want to know what (name of the patient) is telling them, like if you're having a confidential counselling, …I just want them to listen to what I've got to say about how I think he is without telling him, because he says, oh you've been talking to the nurses.*

*(ID 030, caregiver to a son, not 1^st^ admission)*

## Discussion

### Main findings

For many family caregivers, having a relative involuntarily admitted can be a conflicting emotional experience as they frequently feel a sense of relief accompanied by worries and guilt. Family caregivers report difficulties obtaining help when the patient is acutely unwell. They may not know how to initiate contact with services, particularly if the patient has had no previous admissions to a psychiatric hospital. Some think that services focus more on responding to crises than pre-empting them. Family caregivers are concerned that services tend to shift the burden of care to them, and regard confidentiality as a difficult issue.

### Strengths and limitations

The study addressed a clinically important topic that has rarely been explored in research. It included a relatively large number of in-depth interviews with family caregivers of patients admitted to hospitals across different areas of England. All interviews were independently coded by two researchers, and family caregivers were involved in the design of the study and analysis of the results.

A limitation of the study is that there were several potential selection biases in the recruitment of family caregivers - only caregivers of patients who gave consent could have been approached, only 50% of all eligible patients who had been involuntarily admitted to the participating hospitals during the study period agreed to participate in the study and of those who did participate majority were living alone and did not have a family caregiver [16].

However, the recruited sample included family caregivers with a range of relationships to the patient and patients had a range of psychiatric diagnosis, age, ethnicity and living arrangements.

The study was conducted in only one country which may limit the international generalisablity of the results. The interviews were based on family caregivers' recall of events (up to 15 months after the event) and therefore recall bias and discrepancies are likely to occur and present problems in terms of accuracy and reliability [19]. However the accounts of the family caregivers resonate with one another and with the results of other qualitative studies.

### Comparison with literature

Previous research indicates that long term caregiving in mental health is associated with stress and emotions such as shame, embarrassment, feelings of guilt and self-blame [7]. However a feeling of relief in response to the unwell relative's commencement of treatment has not been noted in previous studies and therefore may be contextually specific for acute situations that require compulsory hospital admission.

The findings that mental health services are perceived as poor at proactive involvement and instead mainly responding to crisis confirms results of a study by Jones et al. [20] who interviewed patients and family caregivers and found that the crisis-led nature of services meant that those patients who were relatively stable, or did not display signs of potential risk to self or others felt isolated and on the periphery of service delivery.

Information sharing is important throughout mental health care [21], and as part of good clinical psychiatric practice family caregivers should be involved in the process of assessment and treatment of the patient unless the patient refuses consent to share information. Previous research suggests that general information (such as information about illness and treatment if a diagnosis is known to a family caregiver) can be shared without breaching the confidentiality [22] however for sharing of personal information not known to the carer consent needs to be considered.

### Interpretation

Family caregivers' experience of involuntary hospital admission of their relative is characterised by a strained relationship with mental health services accompanied by practical difficulties in getting help and conflicting emotions experienced in response to their relative's admission.

**Figure 1:**
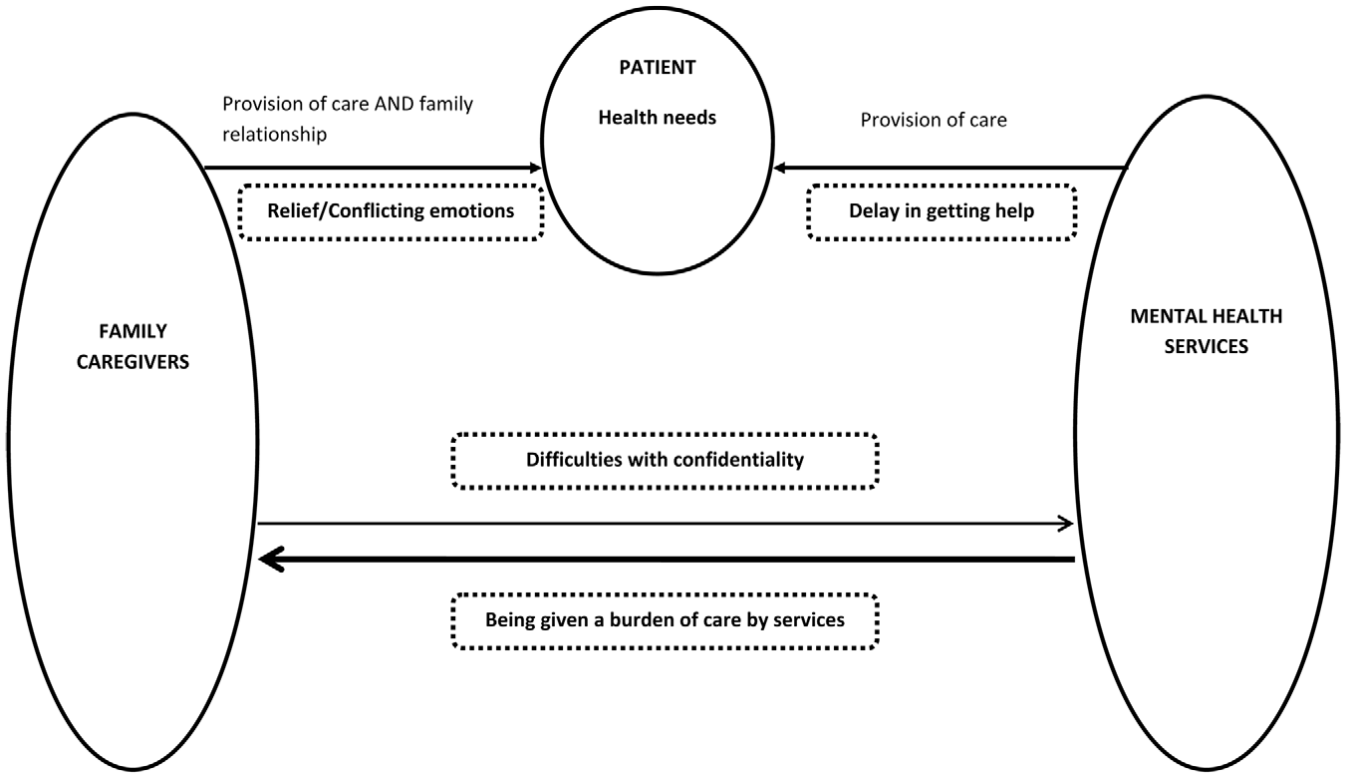
Schematic representation of family caregivers' experiences of involuntary admission of their relative.

Their experiences can be presented schematically within a system (Figure 1).

If mental health services do not provide sufficient care for the patient, the burden on family caregivers and the intensity of care they need to provide increases. This happens when there is a delay in getting help if a situation is not perceived as urgent, when family caregivers do not know how to contact services and when patients are discharged earlier or admitted later (for example only once their mental health significantly deteriorates) without involving caregivers in a decision making process.

Family caregivers and clinicians can have different views on when a hospital admission is required, particularly if this is against the patient's will. For family caregivers this is not just a difference in opinion, but usually also a shift of the responsibility for care towards them which can be experienced as very burdensome.

Caregivers are often in a position in which they perceive that patients' needs (e.g. for a least restrictive treatment outside the hospital) and services' needs (e.g. for a community treatment because of lack of availability of hospital beds and cost of inpatient care) have more priority compared to caregivers' needs when they are finding caring for the patient at home too burdensome. Mental health services do not have an explicit duty of care to the family caregiver whose needs therefore may be ignored or incompletely addressed.

Both family caregivers and mental health services provide care for a patient with mental health problems at the time when he or she is acutely unwell. But whilst mental health services have a professional role, the caregivers' role is intertwined with their family relationship with a patient. This emotionally laden aspect of their involvement can add to the burden of care.

When the pressure on caregivers grows, there is a risk of caregivers' disengagement because of the intensity of the burden and because of the perception that services are dismissing their needs.

Even though results indicating that family caregivers perceive that burden of care is shifted towards them may not come as surprise to clinicians, this has not been shown in previous research and our results add validity to this clinical impression.

When considering mutual influences between a patient, mental health service and a family caregiver, it is important to acknowledge that there is a power difference in the relationship between family caregivers and mental health services as services have much more influence in deciding what level of care patient will receive and who will provide that care.

The tension in the relationship between family caregivers and mental health services is compounded by difficulties with confidentiality which caregivers see as a right that is not always respected. This study highlights the challenge for clinicians to balance two important tasks that at times can be difficult to accommodate: valuing the important role of family caregivers in the treatment process including the sharing of relevant information with them and the requirements of confidentiality with regard to what the patient has told them. This can be particularly difficult before or during an involuntary admission when risks have to be considered which may include specific risks to a family caregiver.

In terms of emotional response, in the acute situations when a patient's mental health deteriorates so much that a hospital admission against their will is required, the burden of care for a family caregivers is extremely high and the feeling of relief once the help is in place is an understandable emotional consequence. However as this help (i.e. admission) is against their relative's will, emotions of worry and guilt often accompany this sense of relief.

In the constellation described above there is a risk of disempowerment of caregivers that in this study is illustrated by experiences that family caregivers' opinion and their needs are not being valued in a decision making process, by lack of information and problems with confidentiality.

### Conclusion

In order for caregivers to be effective partners in care a balance needs to be struck between welcoming and valuing their involvement in providing care for a patient and not overburdening them.

In terms of practical implications mental health professionals need to be aware of a possible tension in their relationship with family caregivers and to be aware of risk of placing excessive demands on them whilst not addressing their needs. Before and during an involuntary admission, family caregivers require timely access to services for the patient and adequate provision of information for themselves. Initiatives such as joint crisis planning involving family caregivers [23] are likely to help them obtain information and find access to services when needed. For caregivers of patients who are experiencing a first episode of illness and who had no previous contact with mental health services, more information needs to be available through primary care and possibly through mental health awareness initiatives. Clinicians should be clear about what information can be shared with the family caregiver and whilst this is not so difficult when the patient consents to sharing the information, best practice guidelines for what happens when the patient does not consent need to be followed and explained to the family caregiver. In addition to these practical steps there are also aspects of staff attitude and clinical practice that may be improved. It is important that clinicians elicit caregivers' views on treatment plans proactively and respect their right to refuse care if it is too burdensome. Improving these attitudes may take time and require more specific training and supervision.

It seems that a decade later, Szmukler's and Bloch's opinion that in the era of community psychiatric care too much is expected from family caregivers but that this has not been balanced by mapping out the duties of services towards them [24] reflect views of the family caregivers interviewed in this study.
